# Atrial Functional Mitral Regurgitation Is a Risk Factor for Permanent Pacemaker Implantation

**DOI:** 10.3390/jcm14238291

**Published:** 2025-11-21

**Authors:** Kyungsub Song, YoHan Bae, Jung Uk Woo, Sungsil Yoon, Hee Jeong Lee, Woo Sung Jang, Yun Seok Kim, Jonghoon Yoo

**Affiliations:** 1 Department of Thoracic and Cardiovascular Surgery, Keimyung University Dongsan Hospital, Keimyung University College of Medicine, Daegu 42601, Republic of Korea; qwlswlstoo@naver.com (Y.B.); sungsilcs@dsmc.or.kr (S.Y.); whiteuri@dsmc.or.kr (W.S.J.); yunseok99@hanmail.net (Y.S.K.); 2 Medical Course, School of Medicine, Keimyung University College of Medicine, Daegu 42601, Republic of Korea; junguk1427@gmail.com; 3 Department of Cardiology, Keimyung University Dongsan Hospital, Keimyung University College of Medicine, Daegu 42601, Republic of Korea; 910827lhj@naver.com; 4 Department of Emergency Medicine, Keimyung University Dongsan Hospital, Keimyung University School of Medicine, Daegu 42601, Republic of Korea; wsnatz@gmail.com

**Keywords:** mitral valve insufficiency, atrial fibrillation, Maze procedure, permanent pacemaker

## Abstract

**Background and Objectives:** This study aimed to investigate risk factors for permanent pacemaker (PPM) implantation following the Maze procedure in patients with atrial functional mitral regurgitation (AFMR). **Methods:** A retrospective cohort of 423 patients who underwent the Maze procedure for persistent or paroxysmal atrial fibrillation from 2010 to 2025 was analyzed. Patients were categorized on the basis of the need for PPM postoperatively. Risk factors and rhythm outcomes were compared using multivariable Cox and logistic regression with backward stepwise selection according to the Akaike Information Criterion. **Results:** Forty-five patients (10.6%) required PPM implantation following the Maze procedure. The PPM group demonstrated a significantly higher AFMR prevalence than the non-PPM group (28.9% vs. 10.4%, *p* = 0.001). Preoperative fine P waves and older age were additional significant predictors. The PPM group exhibited lower postoperative sinus rhythm rates and higher junctional rhythm rates. AFMR (hazard ratio [HR], 2.10; *p* = 0.030), fine P wave (HR, 2.03; *p* = 0.049), and age (HR, 1.04; *p* = 0.018) independently predicted PPM implantation. AFMR particularly elevated the late PPM implantation risk. **Conclusions:** AFMR is an independent risk factor for late nodal dysfunction requiring PPM following Maze procedures. To detect delayed pacemaker requirements postoperatively, extended monitoring is recommended for patients with AFMR.

## 1. Introduction

Functional mitral regurgitation is characterized by regurgitation in the presence of structurally preserved mitral leaflets [1]. It encompasses two major subtypes distinguished by underlying pathophysiologic mechanisms: ventricular functional mitral regurgitation (VFMR) and atrial functional mitral regurgitation (AFMR). VFMR frequently occurs in patients with impaired left ventricular (LV) function and aligns with the Carpentier type III classification. In contrast, AFMR corresponds to Carpentier type I, reflecting regurgitation secondary to atrial dilation [2].

Considerable research has focused on VFMR and its clinical implications [3]; however, studies investigating AFMR-specific surgical outcomes are scarce. The epidemiologic occurrence of AFMR is closely associated with the increasing prevalence of atrial fibrillation (AF) [1], its principal etiologic factor. Significant AFMR, defined as moderate or greater regurgitation, most frequently develops in individuals with persistent AF exceeding 10 years in duration [4]. Published studies have reported that the AFMR prevalence in populations with long-standing AF is approximately 28%.

Current literature highlights the therapeutic value of rhythm control, particularly ablation, in managing patients with AFMR [5]. Although recent evidence suggests favorable operative outcomes in AFMR cohorts [2,6,7], systematic studies delineating Maze procedure outcomes remain lacking [8,9].

Although the sinus rhythm conversion rate following the Maze procedure is crucial, the incidence of permanent pacemaker (PPM) implantation due to atrioventricular block or sick sinus syndrome is also a significant operative outcome following the Maze procedure. The research team previously analyzed the clinical outcomes of the Maze procedure in patients with AFMR, identifying an increased risk of PPM implantation [5,7,8]. The present study aimed to supplement prior surgical data and systematically evaluate the efficacy of the Maze procedure and the risk of PPM implantation in patients with AFMR.

## 2. Materials and Methods

### 2.1. Study Design

This study was a single-center retrospective analysis. It focused on analyzing the risk factors for PPM implantation following the Maze procedure, with particular emphasis on rhythm outcome analysis. Patients were categorized into the following two groups: those who underwent PPM implantation following the Maze procedure and those who did not. A risk factor analysis was performed, with AFMR included as an independent variable in this study.

### 2.2. Study Cohort

This study comprised patients who underwent the Maze procedure for persistent or paroxysmal AF at a single medical center from August 2010 to June 2025. Patients were excluded when they had a PPM implantation history before the Maze procedure, a diagnosis of infective endocarditis at any point, death within 24 h postoperatively, or aged <18 years. In the final cohort, patients were divided into two groups as follows: those who did not require PPM implantation during follow-up (non-PPM group, n = 378) and those who required PPM implantation following the Maze procedure (PPM group, n = 45).

### 2.3. Outcomes

The risk analysis of PPM implantation following the Maze procedure represents the primary outcome of this study. The evaluation of mortality and the risk of cerebral infarction between patients who received PPM implantation and the non-PPM group is the secondary outcome.

### 2.4. AFMR Definition

In this study, the criteria for diagnosing AFMR were established on the basis of recent literature [9]. AFMR was characterized by the presence of structurally normal LV dimensions, including both global and segmental functions, specifically an LV volume index of ≤85 and ≤78 mL/m^2^ for males and females, respectively, and a preserved LV ejection fraction (EF) of ≥50%. Moreover, a diagnosis of AFMR required evidence of significant left atrial (LA) enlargement, as demonstrated by an LA volume index (LAVI) of at least 40 mL/m^2^ and a maximal anteroposterior diameter of ≥40 mm measured during end-systole on M-mode imaging. Furthermore, mitral annular dilation and flattening with unrestricted leaflet mobility (Carpentier type I) were crucial diagnostic features.

### 2.5. Operative Techniques and the Maze Procedure

Here, the surgical protocol encompassed a modified Cox–Maze approach utilizing cryoablation, performed with either antegrade or retrograde cardioplegia per established methodology [10]. Lesion sets for the left atrium were created via three principal ablation lines: two extended from the superior and inferior borders of the left atriotomy toward the LA appendage to facilitate pulmonary vein isolation, and one line was directed from the left atriotomy to the posterior mitral annulus, corresponding to the endocardial mitral isthmus. For the right atrium, ablation lines connected the superior and inferior vena cava, included a T-shaped extension from the intercaval region to the tricuspid annulus, and a separate line ran from the right atriotomy toward the tricuspid annulus through the right atrial appendage. LA appendage exclusion or closure was consistently performed via either internal obliteration or excision based on the attending surgeon’s discretion. The core ablation lesion pattern remained consistent throughout the study, with incremental technical refinements implemented as perioperative experience progressed.

### 2.6. Data Collection and Follow-Up

Clinical variables and outcome measures for this study were systematically collected from the electronic medical record systems of both participating institutions. Early PPM implantation was defined as PPM implantation within the first 30 days following the Maze procedure, whereas late PPM implantation was defined as PPM implantation after 30 days following the Maze procedure. Chronic kidney dysfunction was identified by evidence of renal injury or an estimated glomerular filtration rate of <60 mL/min/1.73 m^2^ sustained for at least 3 months before intervention, regardless of etiology. Fine AF was identified on electrocardiography (ECG) by fibrillatory waves of <0.1 mV across all leads. Preoperative transthoracic echocardiography (TTE) corresponded to results most proximate to the time of surgery.

During the longitudinal follow-up, patients underwent outpatient ECG at 1, 3, and 6 months postoperatively, and at 6-month intervals thereafter. Sinus rhythm was considered restored when at least two consecutive ECGs confirmed its presence. Any arrhythmias, including AF, atrial flutter, ectopic or junctional rhythms, and paced rhythms lacking atrioventricular synchrony, were classified as unsuccessful sinus rhythm restoration. The mean follow-up duration was 56.6 ± 40.1 months for the entire cohort.

Cardiologists specializing in electrophysiology discussed the indication for and timing of postoperative PPM implantation following corresponding guidelines [11].

### 2.7. Statistical Analysis

Continuous variables were compared using the independent t-test and were presented as means ± standard deviations. Categorical variables were expressed as numbers (percentages) and compared using Pearson’s chi-square test or Fisher’s exact test. All tests were two-sided, and a *p*-value of <0.05 was considered statistically significant.

The rhythm outcome differences between the two groups following the Maze procedure were evaluated using a generalized estimating equation model. The repeated rhythm outcomes analyzed encompassed normal sinus rhythm, junctional rhythm, and freedom from AF. ECG data at three time points were used: postoperatively, 1 year postoperatively, and 3 years postoperatively.

PPM implantation was analyzed from three perspectives: early implantation (within 30 days postoperatively), late implantation (>30 days postoperatively), and overall implantation (regardless of timing). Kaplan–Meier survival curves were constructed for each category, and intergroup differences were evaluated using the log-rank test. For overall and late PPM implantation, risk factor analysis for PPM implantation was performed using a multivariate Cox regression model, whereas for early PPM implantation, a multivariate logistic regression analysis was conducted. To construct the multivariable model, we applied a backward stepwise selection method with the Akaike Information Criterion as the criterion for model optimization. The results were reported with corresponding hazard (HR) or odds ratios, 95% confidence intervals (CIs), and *p*-values. Statistical analysis was performed using R version 4.5.0 (R Foundation for Statistical Computing).

## 3. Results

**Figure 1:**
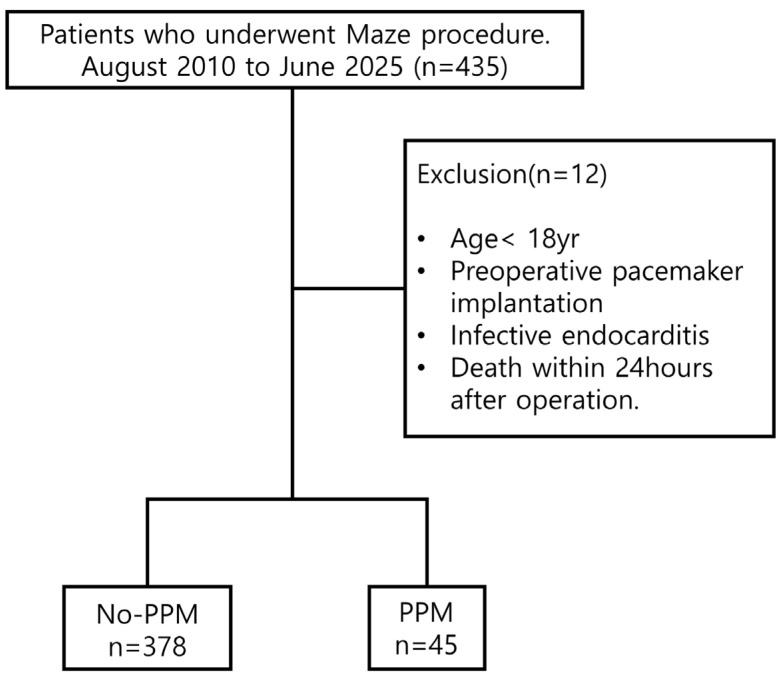
Flow diagram. PPM, permanent pacemaker.

Of the participants, 378 and 45 were included in the non-PPM and PPM groups, respectively (Figure 1). Of the 45 patients who required PPM implantation during follow-up, 9 necessitated early PPM implantation (within 30 days postoperatively) (median, 14 [interquartile range, 7–18] days).

### 3.1. Patient Characteristics

In the overall cohort (Table 1), the PPM group had a significantly higher proportion of patients with AFMR than the non-PPM group (28.9% vs. 10.4%, *p* = 0.001). No significant difference in preoperative AF types was noted between the groups (persistent AF: non-PPM 75.4% vs. PPM 80.0%; paroxysmal AF: non-PPM 24.6% vs. PPM 20.0%; *p* = 0.495). However, the PPM group exhibited a significantly more frequent incidence of AF with fine P waves (73.3% vs. 54.0%, *p* = 0.013). No significant differences were observed between the groups in terms of preoperative comorbidities. Preoperative TTE revealed that the PPM group had significantly higher LVEF and LAVI than the non-PPM group (EF: 57.9% ± 11.4% vs. 52.1% ± 12.5%, *p* = 0.002; LAVI: 135.2 ± 94.2 vs. 107.9 ± 54.7 mL/m^2^, *p* = 0.005).

### 3.2. Operative Results

The operative outcomes are summarized in Table 2. Across the entire cohort, the average follow-up duration following the Maze procedure was 56.6 ± 40.1 months. No significant differences were observed between the two groups in terms of concomitant procedures performed alongside the Maze procedure.

### 3.3. Rhythm Outcomes and Risk Factor Analysis for PPM Implementation Following the Maze Procedure

**Figure 2:**
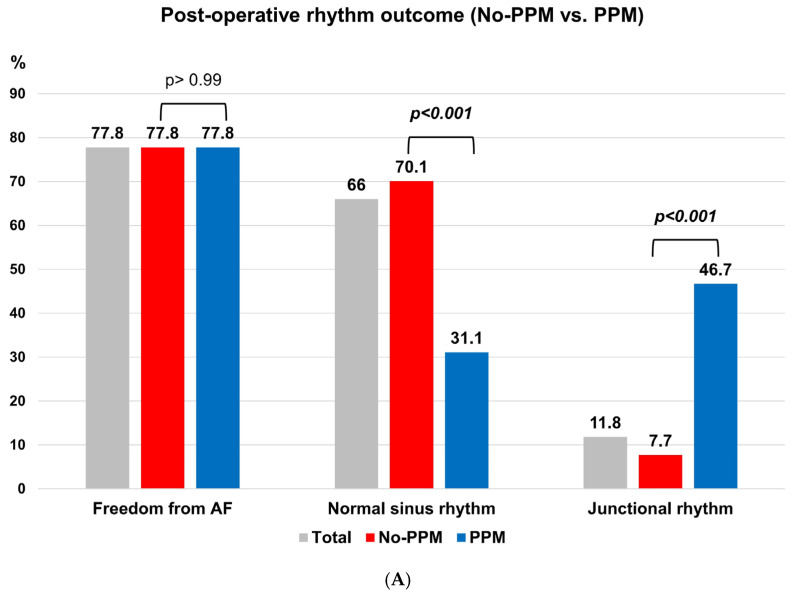

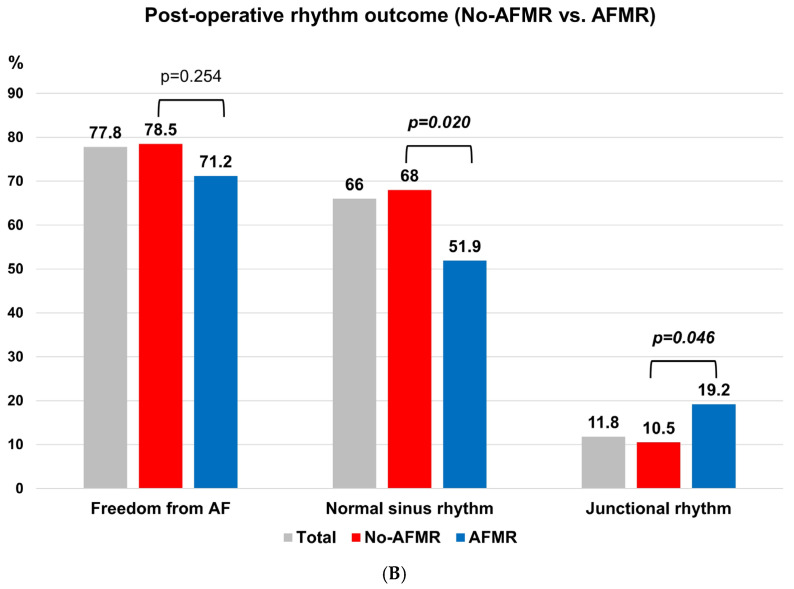
Post-Maze procedure rhythm outcomes. (**A**) Rhythm outcomes in the non-PPM versus PPM groups. In the PPM group, the rate of freedom from atrial fibrillation (AF) is not significantly different between the two groups. However, significantly more patients with junctional rhythm and fewer patients with normal sinus rhythm are noted. (**B**) Rhythm outcomes in the non-AFMR versus AFMR groups. In the AFMR group, the rate of freedom from AF is not significantly different between the two groups. However, significantly more patients with junctional rhythm and fewer patients with normal sinus rhythm are noted. AFMR, atrial functional mitral regurgitation; PPM, permanent pacemaker.

The postoperative rhythm outcomes are summarized in Figure 2. Both groups exhibited a comparable proportion of patients achieving freedom from AF (77.8%, *p* > 0.99). However, the non-PPM group showed a significantly higher normal sinus rhythm incidence than the PPM group (70.1% vs. 31.1%, *p* < 0.001), whereas the PPM group exhibited a significantly more frequent junctional rhythm incidence than the non-PPM group (46.7% vs. 7.7%, *p* < 0.001) (Figure 2A).

Furthermore, we compared postoperative rhythm outcomes between patients without AFMR (non-AFMR) and those with AFMR. The rate of freedom from AF did not significantly differ between both groups (non-AFMR 78.5% vs. AFMR 71.2%, *p* = 0.254). However, patients without AFMR demonstrated a significantly higher normal sinus rhythm incidence than those with AFMR (68.0% vs. 51.9%, *p* = 0.020). In contrast, patients with AFMR exhibited a significantly more frequent junctional rhythm incidence than those without AFMR (19.2% vs. 10.5%, *p* < 0.046) (Figure 2B).

**Figure 3:**
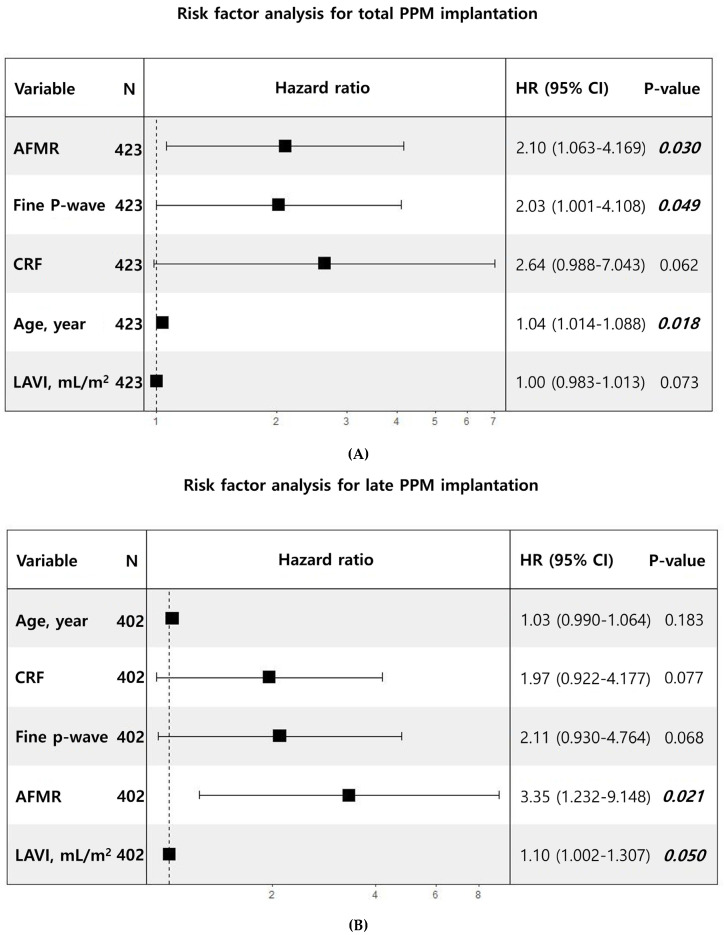

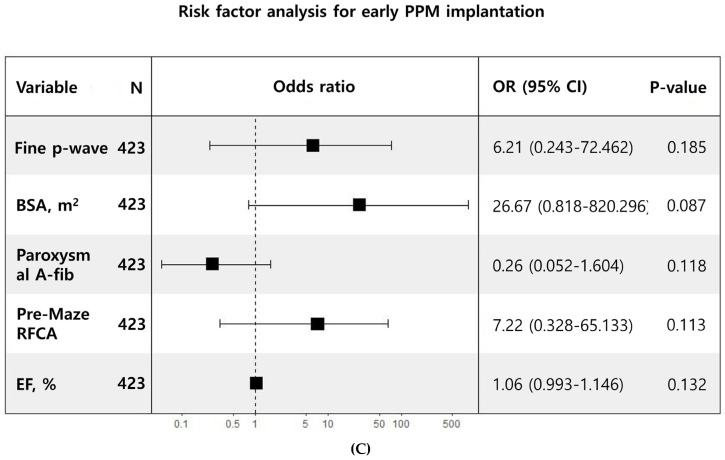
Multivariate risk factor analysis for permanent pacemaker (PPM) implantation. (**A**) Multivariate Cox regression for total PPM implantation. Atrial functional mitral regurgitation (AFMR), pre − Maze fine P wave, and age are identified as significant risk factors for overall PPM implantation, including both early and late PPM implantations. (**B**) Multivariate Cox regression for late PPM implantation. AFMR is the only significant risk factor for late PPM implantation (occurring at >30 days following the Maze procedure). (**C**) Multivariate logistic regression for early PPM implantation. No significant risk factors are noted for early PPM implantation (occurring at <30 days following the Maze procedure). BSA, body surface area; EF, ejection fraction; RFCA, radiofrequency catheter ablation. The black square represents the odds ratio.

Of the 45 patients in the PPM group, 9 underwent early PPM implantation (within 30 days postoperatively), whereas 36 necessitated late PPM implantation (>30 days following the Maze procedure). Cox multivariate analysis for total PPM implantation (including both early and late cases) identified AFMR (HR, 2.10; 95% CI, 1.063–4.169; *p* = 0.030), preoperative fine P wave (HR, 2.03; CI, 1.001–4.108; *p* = 0.049), and age (HR, 1.04; 95% CI, 1.014–1.088; *p* = 0.018) as significant risk factors (Figure 3A). In the risk factor analysis for late PPM implantation (Figure 3B), AFMR was the only significant predictor (HR, 3.35; 95% CI, 1.232–9.148; *p* = 0.021). No significant risk factors were identified for early PPM implantation (Figure 3C).

**Figure 4:**
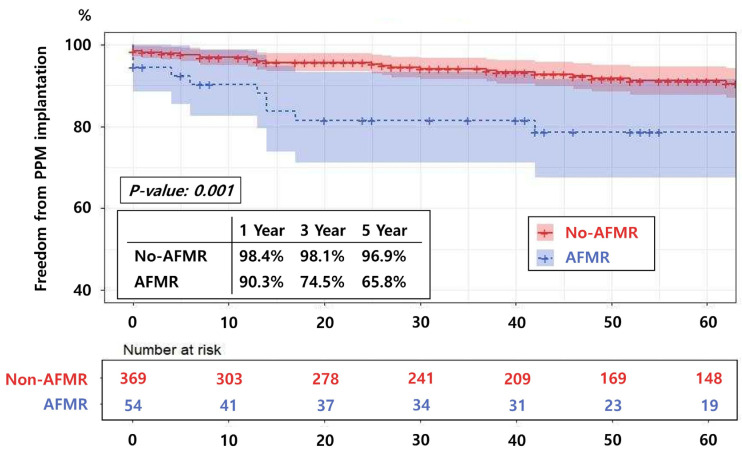
Kaplan–Meier analysis of freedom from permanent pacemaker (PPM) implantation in patients without atrial functional mitral regurgitation (non-AFMR) versus those with AFMR. Patients with AFMR demonstrate a significantly higher PPM implantation incidence. AFMR, atrial functional mitral regurgitation.

For both overall and long-term PPM implantations, AFMR was identified as a significant risk factor. Subanalysis comparing the AFMR and non-AFMR groups in terms of long-term outcomes of PPM implantation revealed that the rates of freedom from PPM implantation at 1, 3, and 5 years following the Maze procedure were 98.4%, 98.1%, and 96.9% in the non-AFMR group, and 90.3%, 74.5%, and 65.8% in the AFMR group, respectively (Figure 4) (*p* = 0.001).

### 3.4. Long-Term Survival Analysis

**Figure 5:**
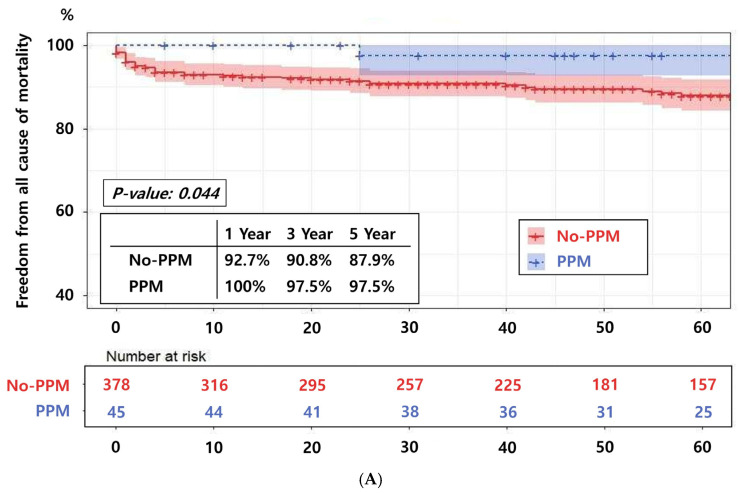

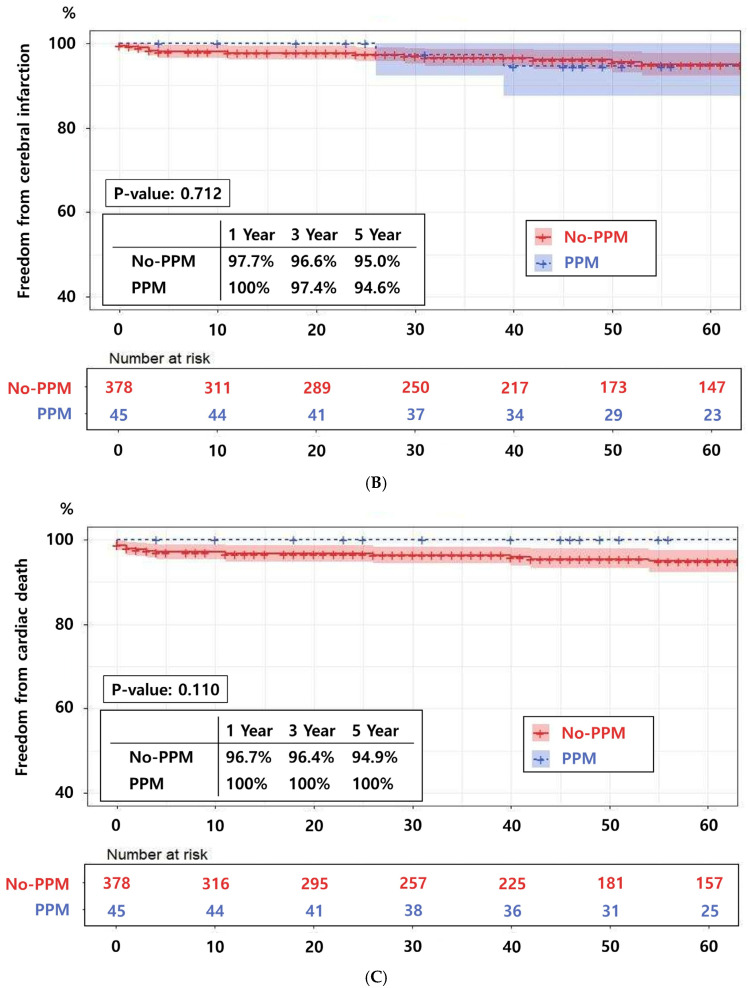
Kaplan–Meier analysis of the rates of all-cause mortality, cerebral infarction, and cardiac death in patients who did not require a permanent pacemaker (non-PPM) versus those who required a permanent pacemaker (PPM). No significant differences in all-cause mortality (**A**), cerebral infarction (**B**), or cardiac death (**C**) are observed between the non-PPM and PPM groups.

The 1-, 3-, and 5-year survival rates for the non-PPM and PPM groups were 92.7%, 90.8%, and 87.9% versus 100%, 97.5%, and 97.5%, respectively (*p* = 0.044), indicating a significantly higher survival rate in the PPM group (Figure 5A). However, no significant differences were noted between the non-PPM and PPM groups in terms of freedom from cardiac death and stroke rates at 1, 3, and 5 years: 97.7%, 96.6%, and 95.0% versus 100%, 97.4%, and 94.6% (*p* = 0.712) for cardiac death, respectively (Figure 5B), and 96.7%, 96.4%, and 94.9% vs. 100%, 100%, and 100% (*p* = 0.110) for stroke, respectively (Figure 5C).

## 4. Discussion

AFMR being identified as a significant risk factor for PPM implantation following the Maze procedure, particularly increasing the risk of late PPM implantation, is the main finding of this study. This finding represents a crucial discovery in AFMR, for which data regarding outcomes following the Maze procedure are limited.

Previous studies have focused on the prognosis of AFMR following mitral valve repair [2,6,7], specifically regarding mitral regurgitation-related mortality and recurrence rates. However, despite the significance of the Maze procedure in AFMR, research on the outcomes of the Maze procedure in patients with AFMR is currently scarce. In our previous multicenter and single-center retrospective studies [7,8], we have reported that patients with AFMR demonstrated a higher postoperative junctional rhythm incidence following the Maze procedure than those with other valvular diseases. The reason is that patients with AFMR tend to have a longer AF duration, potentially increasing concomitant sinus nodal dysfunction.

In particular, the occurrence of junctional rhythm following the Maze procedure in patients with AFMR significantly increased the risk of PPM implantation. The high junctional rhythm prevalence and elevated PPM implantation risk in patients with AFMR may be attributed to the extended AF duration and consequent sinus nodal dysfunction [12,13,14,15,16,17,18,19]. Previous studies have revealed that a longer AF duration causes progressive sinus nodal dysfunction; following the Maze procedure, previously masked sinus nodal dysfunction may become unmasked, leading to junctional rhythm. Similarly, in cases of radiofrequency catheter ablation (RFCA), prolonged AF duration, sinus nodal dysfunction, bradycardia, or elevated LAVI have all been reported to increase the risk of junctional rhythm and PPM implantation following RFCA [20,21,22]. These are all distinctive features frequently observed in patients with AFMR.

In this study, AFMR was not identified as a significant risk factor for early PPM implantation; however, it was noted to be a major risk factor for late PPM implantation. Previous studies have indicated that in patients with AF, longer AF duration before RFCA or Maze procedure is associated with ongoing structural and electrical remodeling of the atria as well as progressive atrial fibrosis, even following ablation therapy [23,24,25]. Consequently, this incident results in a higher risk of sinus node dysfunction, elevating the probability of subsequent PPM implantation. Similarly, an increased risk of late rather than early PPM implantation was observed in patients with AFMR, suggesting that nodal dysfunction in this population gradually progresses following the Maze procedure. Therefore, the elevated risk of late PPM implantation highlights the importance of extended monitoring and risk stratification in this subgroup. Recent references also support preoperative counseling regarding the risk of PPM implantation in AFMR patients, and recommend ongoing follow-up to promptly identify delayed nodal dysfunction [26,27].

Additionally, recent developments in cardiac pacing, such as conduction system pacing (CSP) and cardiac resynchronization therapy (CRT), have important implications for Maze patients, particularly those with AFMR [28]. CSP targets the His bundle or left bundle branch, preserving normal electrical activation and reducing dyssynchrony. This approach is especially relevant for AFMR patients, who are prone to conduction abnormalities following the Maze procedure. Conventional right ventricular pacing can worsen ventricular dyssynchrony and increase the risk of heart failure or mitral regurgitation [29,30]. Studies show that CSP improves synchrony, left ventricular function, and may lower the risk of pacing-induced cardiomyopathy. Similarly, CRT enhances ventricular function and can reduce the severity of mitral regurgitation by optimizing timing and activation [31]. These approaches are likely to provide significant benefits for patients with AFMR, who frequently experience atrioventricular block after the Maze procedure. In particular, they may help reduce the recurrence rate of mitral regurgitation following mitral valve repair in patients with AFMR, as CSP and CRT have been shown to more effectively prevent the worsening of mitral regurgitation compared to conventional PPM.

In this study, there was no significant difference in the AF recurrence rate after the Maze procedure between patients with AFMR and those without AFMR (71.2% vs. 78.5%, *p* = 0.254). This may be because the mechanisms of post-Maze procedure AF recurrence and atrial flutter (AFL) do not differ substantially between AFMR and non-AFMR patients. Generally, a significant proportion of Maze patients experience atrial arrhythmias such as recurrent AF or atrial flutter (AFL) even after surgical ablation. According to Wazni et al. [32], about one-third of patients have recurrent AF due to recovery of conduction pathways between the pulmonary veins and the left atrium. Incisional AFL is another common postoperative arrhythmia, resulting from macro-reentrant circuits created by incomplete ablation lines or scar tissue formation. Persistent or late postoperative arrhythmias are often caused by tissue remodeling, progressive fibrosis, and reconnection of previously isolated zones.

The subanalysis of patients who underwent PPM implantation revealed promising results for mortality and stroke risks. Although the PPM group exhibited a significantly better all-cause mortality rate than the non-PPM group, this finding may be due to the limitations of a small cohort study. Overall, these results suggest an acceptable long-term mortality and stroke risks in patients with PPM implantation.

This study had several limitations. First, this study was not a randomized controlled trial, and the data were retrospectively analyzed in a limited number of cohorts. Second, follow-up screening for AF recurrence was based on serial 12-lead ECG rather than a 24-h Holter monitoring. Therefore, our freedom from AF results may have been overestimated because asymptomatic paroxysmal AF may have been present during the follow-up period without appearing on subsequent ECGs performed in outpatient clinics. Third, this study did not include an analysis of advanced imaging findings. Emerging evidence suggests that speckle-tracking echocardiography derived left atrial reservoir strain (LASr) is a highly sensitive marker of left atrial fibrotic remodeling [33]. Because LASr impairment may independently predict the risk of permanent pacemaker implantation even in the absence of AFMR [34], future studies should investigate whether LASr measurement can further improve risk stratification beyond conventional echocardiographic parameters.

In conclusion, this study identified AFMR as a major risk factor for sinus nodal dysfunction and subsequent PPM implantation following the Maze procedure. Notably, since this nodal dysfunction tends to progress over time even following the Maze procedure, close monitoring is warranted in patients without AF following the Maze procedure.

## Figures and Tables

**Table 1:** Patients’ characteristics.

	Total (n = 423)	Non-PPM (n = 378)	PPM (n = 45)	*p*-Value
Sex, male, (%)	211 (49.9)	190 (50.3)	21 (46.7)	0.648
Age, years	63.8 ± 9.4	63.6 ± 9.6	65.4 ± 7.8	0.224
Body surface area, m^2^	1.7 ± 0.2	1.7 ± 0.2	1.7 ± 0.2	0.656
AFMR, (%)	54 (12.8)	41 (10.4)	13 (28.9)	***0.001***
AF type, (%)				0.495
Persistent	321 (75.9)	285 (75.4)	36 (80.0)	
Paroxysmal	102 (24.1)	93 (24.6)	9 (20.0)	
Fine P wave, (%)	237 (56.0)	204 (54.0)	33 (73.3)	***0.013***
Comorbidity, (%)				
Heart failure	144 (34.0)	129 (34.1)	15 (33.3)	0.915
Previous cardiac surgery	21 (5.0)	19 (5.0)	2 (4.4)	1.000
Coronary artery disease	41 (9.7)	37 (9.8)	4 (8.9)	1.000
Cerebral infarction	68 (16.1)	59 (15.6)	9 (20.0)	0.448
Peripheral artery disease	5 (1.2)	5 (1.3)	0	1.000
Rheumatic heart disease	28 (6.6)	28 (7.4)	0	0.059
Diabetes mellitus	89 (21.0)	79 (20.9)	10 (22.2)	0.837
Hypertension	172 (40.7)	156 (41.3)	16 (35.6)	0.461
Pre-Maze RFCA	11 (2.6)	10 (2.6)	1 (2.2)	1.000
CRF	22 (5.2)	17 (4.5)	5 (11.1)	0.072
CRF on hemodialysis	6 (1.4)	6 (1.6)	0	1.000
Preoperative TTE				
Ejection fraction, %	52.7 ± 12.1	52.1 ± 12.5	57.9 ± 11.4	***0.002***
LVESD, mm	3.8 ± 0.7	3.71 ± 0.8	3.5 ± 0.8	0.130
LVVI, mL/m^2^	62.1 ± 25.5	63.1 ± 26.8	57.7 ± 18.5	0.099
LAVI, mL/m^2^	105.1 ± 48.5	107.9 ± 54.7	135.2 ± 94.2	***0.005***

Data are presented as numbers (%) or means ± standard deviations. Significant *p*-values are shown in italics and bold. The left atrial volume index is measured using left atrial or ventricular volume obtained using Simpson’s method from a four-chamber apical view, indexed to the body surface area. AF, atrial fibrillation; AFMR, atrial functional mitral regurgitation; CRF, chronic renal failure; LAVI, left atrial volume index; LVESD, left ventricular end-systolic diameter; LVVI, left ventricular volume index; PPM, permanent pacemaker; RFCA, radiofrequency catheter ablation; TTE, transthoracic echocardiography.

**Table 2:** Operative results.

	Total (n = 423)	Non-PPM (n = 378)	PPM (n = 45)	*p*-Value
Follow-up period, months	56.6 ± 40.1	54.7 ± 39.6	73.0 ± 41.2	***0.006***
Postoperative hospital stay, days	12.3 ± 31.4	12.1 ± 32.9	13.9 ± 14.5	0.513
Mechanical valve insertion, (%)	98 (23.2)	91 (24.1)	7 (15.6)	0.200
Concomitant operation, (%)				
LAAO, (%)	221 (52.2)	195 (51.6)	26 (57.8)	0.432
Mitral valve replacement	154 (36.4)	139 (36.8)	15 (33.3)	0.650
Mitral valve repair	127 (30.0)	112 (29.6)	15 (33.3)	0.608
Tricuspid annuloplasty	214 (50.6)	189 (50.0)	25 (55.6)	0.481
Aortic valve replacement	76 (18.0)	71 (18.8)	5 (11.1)	0.303
Aortic valve repair	6 (1.4)	6 (1.6)	0	1.000
Coronary artery bypass graft	39 (9.2)	37 (9.8)	2 (4.4)	0.410
Aorta replacement	18 (4.3)	18 (4.8)	0	0.239
Atrial septal defect	43 (10.2)	36 (9.5)	7 (45.6)	0.206
Myxoma removal	3 (0.8)	3 (0.8)	0	1.000
Bleeding control, (%)	16 (3.8)	14 (3.7)	2 (4.4)	0.683

Data are presented as numbers (%) or means ± standard deviations. Significant *p*-values are shown in italics and bold. LAAO, left atrial appendage occlusion; PPM, permanent pacemaker.

## Data Availability

The data presented in this study are available on request from the corresponding author due to the data are not publicly available due to privacy or ethical restrictions.
